# Effects of combined stroboscopic glasses and reaction light training on visual abilities and sport-specific performance in collegiate male volleyball players

**DOI:** 10.1186/s13102-026-01957-6

**Published:** 2026-08-07

**Authors:** Xiushen Dong, Dongxu Gao, Tianci Hou, Congcong Weng, Yuqing Gao, Lei Han, Enmei Bi

**Affiliations:** 1 School of Physical Education, Hunan Normal University, Changsha, China; 2 Division of Sports Science and Physical Education, Tsinghua University, Beijing, 100084 China; 3 Department of Physical Education, Jiaxing University, Jiaxing, China; 4 School of Physical Education, Wuhan Sports University, Wuhan, 430079 China

**Keywords:** Stroboscopic visual training, Reaction light training, Wearable technology, Sensing technology, Volleyball, Visual abilities, Sport-specific performance, Perceptual-motor training

## Abstract

**Objective:**

This study examined the effects of combined stroboscopic glasses and reaction-light training on visual abilities and sport-specific performance in collegiate male volleyball players.

**Methods:**

In this randomized controlled trial, 26 collegiate male volleyball players were assigned to an experimental group (EG, *n* = 13) or an active-control group (CG, *n* = 13). Both groups maintained their regular volleyball training. Over 8 weeks, the EG completed combined stroboscopic and reaction-light training, whereas the CG performed duration- and volume-matched volleyball-specific tasks under normal visual conditions. Training was conducted three times per week for 30 min per session. Reaction time, eye–hand coordination, decision-making ability, coordination quality, agility, and defensive reception success rate were assessed before and after the intervention. Intervention effects were evaluated using two-way repeated-measures ANOVA, with improvement-score analyses and baseline-adjusted ANCOVA conducted as sensitivity analyses. Bonferroni correction was applied to secondary and exploratory outcomes.

**Results:**

Defensive reception success rate showed a significant Group × Time interaction, F(1, 24) = 13.053, *p* = 0.001, partial η² = 0.352, with a between-group difference in change of 3.50% points (95% CI, 1.50–5.50). After Bonferroni correction, significant interactions remained for reaction time (adjusted *p* < 0.001, partial η² = 0.858), eye–hand coordination (adjusted *p* = 0.002, partial η² = 0.423), decision-making ability (adjusted *p* = 0.030, partial η² = 0.279), and coordination quality (adjusted *p* < 0.001, partial η² = 0.827). Agility was not significant after correction (adjusted *p* = 0.110).

**Conclusion:**

Compared with matched volleyball-specific active-control training under normal visual conditions, combined stroboscopic glasses and reaction-light training may provide additional benefits for improving selected visual abilities and defensive reception performance in collegiate male volleyball players. The agility finding should be interpreted cautiously.

**Trial registration:**

ISRCTN Registry ISRCTN10147263, registered on 27 May 2026. Retrospectively registered.

**Supplementary Information:**

The online version contains supplementary material available at https://doi.org/10.1186/s13102-026-01957-6.

## Introduction

Volleyball is a sport with high temporal and spatial constraints, requiring significant perceptual and cognitive demands. Especially in receiving serves and defensive situations, athletes must quickly extract cues from the incoming ball, assess the situation, make decisions, and execute actions within a very short time frame [1, 2]. As a result, volleyball places high demands on visual abilities. Existing research has shown that the visual capabilities of volleyball players are closely related to their performance in the sport [3]. Higher levels of visual processing ability help athletes respond more effectively and control their actions in fast-paced, dynamic game environments [4, 5].

However, research on visual interventions in volleyball remains somewhat limited. Firstly, most studies focus on outcome measures such as reaction time and eye-hand coordination, which are closely related to visual ability [6], with relatively few examining whether these improvements can transfer to sport-specific performance. Secondly, existing intervention methods predominantly rely on single training modalities [7–9], with common approaches including either strobe visual training or reaction light training in isolation. There is less emphasis on the combined impact of these different training methods on overall volleyball performance.

Stroboscopic visual training (SVT) involves intermittent visual input blockage, reducing the availability of continuous visual feedback. This process encourages athletes to rely more on predictive processing, attention distribution, and the extraction of key information to complete tasks [10, 11]. Previous studies have shown that SVT can improve athletes’ visual functions and related performance. In volleyball, six weeks of SVT has been found to enhance visual-motor functions and improve reaction agility [7, 8]. Some studies also suggest that SVT may positively impact defensive performance [12]. Additionally, systematic reviews and meta-analyses further support the beneficial effects of SVT on cognitive function and certain motor performances [13].

Reaction light training, on the other hand, is a perceptual-motor training method in which athletes quickly identify random light signals and react accordingly. This training typically uses programmable light devices to present visual stimuli at different spatial locations, prompting athletes to complete a fast “stimulus recognition—action output” coupling process. Its primary mechanism is to enhance visual information processing efficiency, shorten reaction transition time, and improve visual-motor coordination, thereby improving perceptual-motor performance [12, 14, 15]. Previous research has shown that reaction light training can improve athletes’ visual performance and some sport-specific skills in volleyball [6]. It has also been proven effective in enhancing visual reaction capabilities and specialized skills in other fast-paced sports, such as basketball [14, 16, 17]. Relevant reviews also highlight that visual stimulation-based training generally improves reaction performance and visual-motor integration [18].

The combined training approach used in this study refers to the integration of SVT and reaction light training. Previous research and reviews suggest that SVT can be used not only as an independent training method but also as an additional module embedded into traditional or other training tasks to achieve broader training benefits [19, 20]. In terms of their effects, the focus of the two training methods is not entirely the same. SVT is primarily aimed at improving visual processing, visual-motor integration, and reaction time [11, 21], while reaction light training, in addition to enhancing visual-motor reactions, typically leads to improvements in speed, agility, and certain specialized motor skills [6, 16, 22]. Therefore, combining these two approaches is expected to complement each other, resulting in a more comprehensive training effect. This combined approach is particularly important for volleyball defense. Defenders must not only rapidly identify and process cues from the incoming ball but also respond, move, and control their actions within a very short time frame. This requires both efficient visual processing and strong perceptual-motor coupling. While previous studies have supported the positive effects of SVT and reaction-light training on volleyball players’ visual abilities and some sport-specific performance outcomes, there is still a lack of research that integrates both methods into a single intervention framework and simultaneously examines whether improvements in visual abilities can transfer to sport-specific performance. Based on this, constructing a combined visual training model for sports presents clear research value and practical significance.

Given the analysis above, this study focuses on collegiate male volleyball players and develops a combined visual training program incorporating both SVT and reaction light training. It systematically explores their impact on visual abilities and sport-specific performance. In addition to assessing visual ability indicators such as reaction time, eye–hand coordination, and decision-making, this study further includes sport-specific performance outcomes such as coordination quality, agility, and defensive reception success rate to more comprehensively evaluate the training effect.

In this study, the term visual abilities refers to the laboratory-based visual performance measures, including reaction time, eye-hand coordination, and decision-making ability. The term sport-specific performance refers to volleyball-related performance outcomes, including coordination quality, agility, and defensive reception success rate. The term visual-motor processing is used only when discussing possible perceptual-motor mechanisms, rather than as a separate outcome category. We hypothesized that, compared with matched volleyball-specific active-control training under normal visual conditions, the combined stroboscopic and reaction-light training would produce greater improvements in visual abilities and selected sport-specific performance outcomes.

## Materials and methods

### Study design

**Fig. 1:**
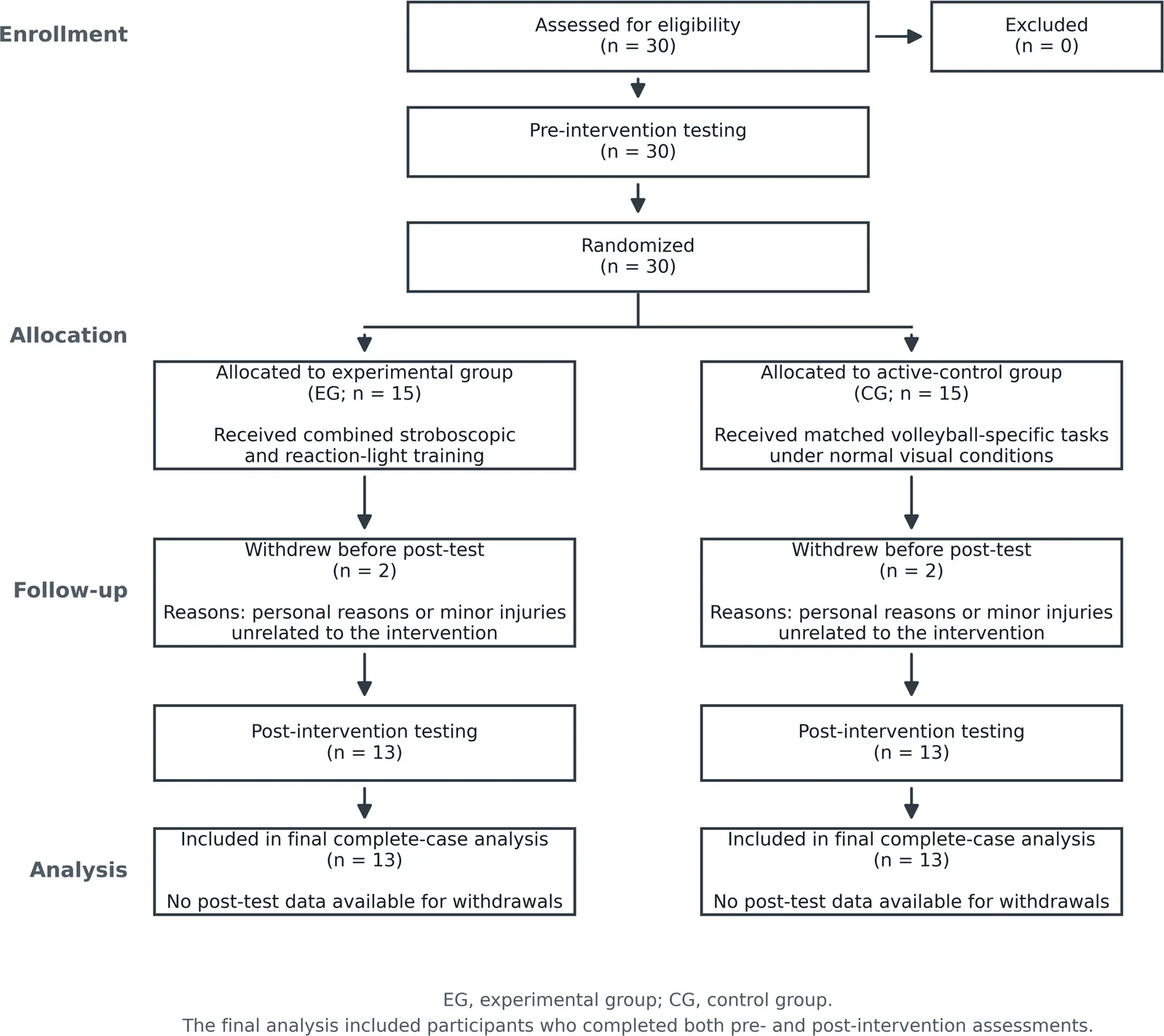
CONSORT flow diagram of participant recruitment, allocation, follow-up, and analysis

This study employed a randomized controlled pre-post experimental design to investigate the effects of combined stroboscopic visual training and reaction light training on visual abilities and sport-specific performance in collegiate male volleyball players. After baseline testing, participants were randomly allocated to the experimental group or the control group in a 1:1 ratio using a computer-generated random number sequence. The random allocation sequence was generated by an independent researcher who was not involved in participant recruitment, intervention delivery, or outcome assessment. To maintain allocation concealment, group assignments were placed in sequentially numbered, opaque, sealed envelopes and were opened only after completion of baseline testing. Another researcher then assigned participants to the groups according to the allocation sequence. The experimental group completed the combined stroboscopic and reaction-light training, whereas the control group completed matched volleyball-specific active-control tasks under normal visual conditions, without stroboscopic occlusion or reaction-light stimuli. The intervention lasted for 8 weeks, with 3 sessions per week, each lasting 30 min. Visual ability and sport-specific performance assessments were conducted at baseline and after the 8-week intervention.The participant recruitment, allocation, follow-up, and analysis process is shown in Fig. 1. This randomized controlled trial was reported in accordance with the CONSORT guidelines. A completed CONSORT checklist is provided as an additional file.

### Participants

A total of 30 collegiate male volleyball players were recruited and randomized, with 15 participants allocated to the experimental group and 15 to the active-control group. During the intervention period, four participants withdrew before post-intervention testing. Specifically, two participants withdrew from the experimental group and two withdrew from the active-control group because of personal reasons or minor injuries unrelated to the intervention. Therefore, 26 participants completed both pre- and post-intervention assessments and were included in the final analysis, with 13 participants in each group. All participants were members of the men’s volleyball team at Hunan Normal University, with an average age of 20.4 ± 1.3 years. They all had a history of systematic volleyball training and maintained regular training during the study. The inclusion criteria were: (1) aged 18–25 years; (2) official members of a collegiate volleyball team; (3) normal vision or vision corrected to normal; and (4) willingness to participate in the intervention and complete pre- and post-intervention assessments. The exclusion criteria were: (1) history of concussion, vestibular dysfunction, or sports injury within the past 3 months; (2) any neurological, visual, or balance disorders. The baseline characteristics of the participants are presented in Table 5. This study was approved by the Ethics Committee of Hunan Normal University (2026264), and all participants provided written informed consent prior to participation.

### Experimental procedure

After recruitment and screening, participants underwent baseline testing, followed by random group assignment and an 8-week intervention phase. Post-intervention testing was conducted after the 8-week intervention period. Both pre- and post-intervention assessments included visual ability tests and sport-specific performance tests. The visual ability tests included reaction time, eye-hand coordination, and decision-making ability, whereas the sport-specific performance tests included coordination quality, agility, and defensive reception success rate.

To minimize measurement error, all tests were conducted at the same venue, under similar time conditions, and according to standardized procedures. Before formal testing, all participants completed a standardized warm-up to reduce the potential influence of fatigue and environmental variation.

Because of the visible nature of the intervention, participant and trainer blinding was not feasible. However, outcome assessors were not involved in intervention delivery and were not informed of participants’ group allocation during post-intervention testing and scoring. Coordination quality was automatically recorded by the FITLIGHT system as average response time and therefore did not involve manual scoring or assessor judgment. Defensive reception success rate was the only observer-dependent sport-specific outcome; therefore, it was video-recorded and evaluated by blinded assessors according to standardized criteria to reduce potential detection bias.

During the study, both groups maintained consistent regular volleyball training. In addition, both groups participated in the scheduled intervention sessions: the experimental group performed the combined visual training, whereas the control group performed matched volleyball-specific active-control tasks under normal visual conditions. The intervention protocol was developed with reference to previous stroboscopic visual training studies [7, 8, 12, 23] and reaction-light or light-based perceptual–motor training studies [6, 14, 16–18]. These previous approaches informed the intervention duration, progressive training structure, visual-stimulus settings, and selection of volleyball-specific defensive tasks.

### Intervention protocol

The intervention lasted 8 weeks, with three 30-min sessions conducted each week. Both groups continued their regular volleyball training throughout the study. In addition, the experimental group completed combined visual training using stroboscopic eyewear and reaction-light stimuli, whereas the active-control group completed matched volleyball-specific tasks under normal visual conditions without stroboscopic occlusion or reaction-light stimuli.

The duration, movement routes, task content, and overall training exposure were matched between groups. Therefore, the primary difference between the groups was the mode of visual stimulus presentation. Each intervention session comprised approximately 15 min of reaction-light training and 15 min of stroboscopic visual training.

#### Reaction-light training

Reaction-light training was conducted using the ReactionX system, which consists of wireless RGB LED targets controlled through a mobile application. The system supports the connection of multiple light targets and allows the activation sequence, stimulus interval, response window, and light color to be customized.

The reaction-light component was adapted from previous light-based perception–action training protocols used in volleyball and other team sports [6, 14, 15, 17]. Based on these approaches and the perceptual–movement demands of volleyball defense, the present protocol progressed from stationary hand- or foot-touch responses to short-distance footwork, multidirectional movement, rapid positioning, and volleyball-specific defensive actions. During the later phases, athletes moved in response to randomly activated targets before performing forearm passing or defensive reception.

**Fig. 2:**
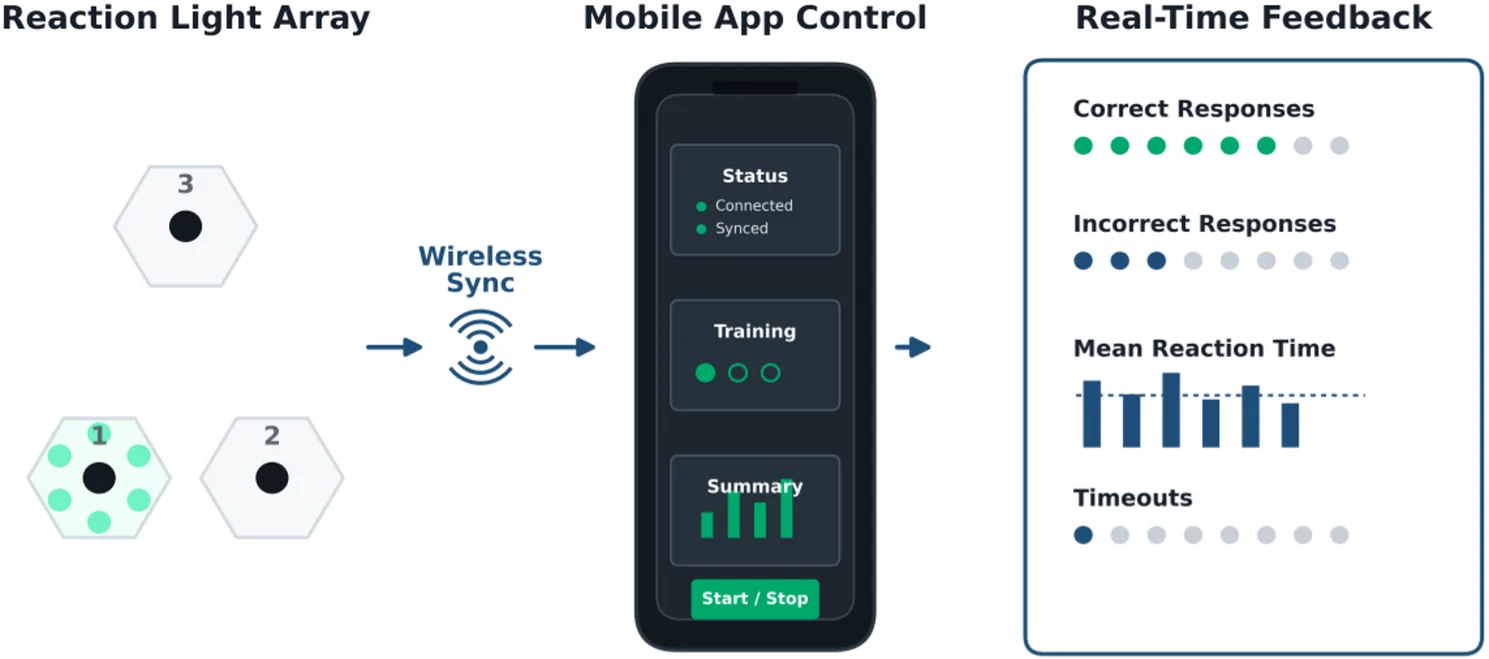
ReactionX reaction-light training system

The active-control group completed the same movement routes, footwork patterns, and volleyball-specific actions. However, cues were provided through standardized coach commands, hand signals, or fixed markers instead of reaction-light stimuli. The ReactionX system is illustrated in Fig. 2, and the detailed training protocol is presented in Table 1.

**Table 1:** Reaction-light training protocol

Phase weeks	Training task	Light arrangement	Stimulus setting	Training dose and progression
1 (1–2)	Stationary hand/foot touch	4 lights in fixed positions, 0.8 m apart	Random activation; 2.0-s response window; 1.0-s interval	3 × 4 min; 1.5-min rest; adaptation to random stimuli
2 (3–4)	Short-distance footwork and target touch	6 lights, 1.0 m apart	Random activation; 2.0-s response window; 0.8-s interval	3 × 4 min; 1.5-min rest; increased light number and stimulus density
3 (5–6)	Multidirectional movement with volleyball footwork	8 lights within a 3 × 3 m area, approximately 1.5 m apart	Random activation; 1.8-s response window; 0.6-s interval	3 × 4 min; 1.5-min rest; increased movement distance and randomness
4 (7–8)	Rapid positioning and defensive actions following light cues	8 lights distributed within the defensive area, 1.5–2.0 m apart	Random activation; 1.5-s response window; 0.4-s interval	3 × 4 min; 1.5-min rest; increased time pressure and task specificity

Each reaction-light component comprised three 4-min sets separated by 1.5 min of rest, totaling 15 min. Stimuli were presented continuously; therefore, the number of responses was not fixed and was recorded automatically by the ReactionX application

#### Stroboscopic visual training

Stroboscopic visual training was conducted using Senaptec Strobe Classic eyewear (Senaptec, Beaverton, OR, USA). The liquid-crystal lenses alternated between transparent and opaque states, intermittently restricting visual information during task performance.

The training content was developed from previous stroboscopic visual training protocols [12, 23] and incorporated volleyball-specific visual–motor tasks. These included stationary passing, receiving standardized tosses, passing after short-distance movement, ball recognition, trajectory judgment, rapid positioning, and defensive reception. The experimental group performed these tasks under intermittent visual-occlusion conditions, whereas the active-control group completed the matched tasks under normal visual conditions.

**Fig. 3:**
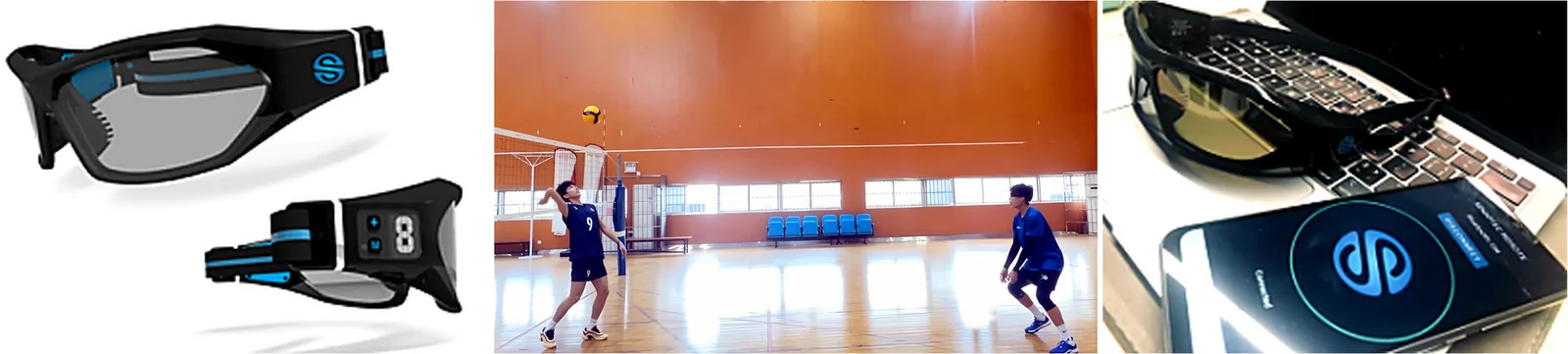
Senaptec Strobe Classic eyewear and volleyball-specific training setup

The stroboscopic device and training setup are illustrated in Fig. 3, and the detailed protocol is presented in Table 2.

**Table 2:** Stroboscopic visual training protocol

Phase weeks	Training task	Strobe and occlusion setting	Training dose	Progression
1 (1–2)	Stationary passing and receiving tossed balls	Mode A; Level 1;	3 × 4 min; 1.5-min rest	Adaptation to intermittent vision
2 (3–4)	Passing after short-distance movement and ball recognition	Mode A; Level 2;	3 × 4 min; 1.5-min rest	Increased movement and visual restriction
3 (5–6)	Reception following multidirectional footwork and defensive movement	Mode A; Level 3;	3 × 4 min; 1.5-min rest	Added directional uncertainty and trajectory judgment
4 (7–8)	Defensive reception after rapid positioning under time pressure	Mode A; Level 4;	3 × 4 min; 1.5-min rest	Highest visual restriction and task specificity

Each stroboscopic component comprised three 4-min sets separated by 1.5 min of rest, totaling 15 min. Mode A involved simultaneous intermittent occlusion of both lenses. Stroboscopic training intensity was controlled using the device’s Mode A and Level 1–4 settings

#### Progression and adherence

Both training components followed a predefined four-phase progression, with training difficulty increased every 2 weeks by increasing the strobe level, number and spatial distribution of light targets, movement distance, stimulus randomness, and task specificity, while shortening the response window and stimulus interval. Progression was permitted when participants completed the prescribed tasks safely, maintained correct volleyball technique, and reported no visual discomfort.

All sessions were supervised by the research team, and attendance and task completion were recorded. Adherence was calculated as the number of completed sessions divided by the 24 scheduled sessions and multiplied by 100.

### Assessment methods

#### Visual ability assessment

**Fig. 4:**
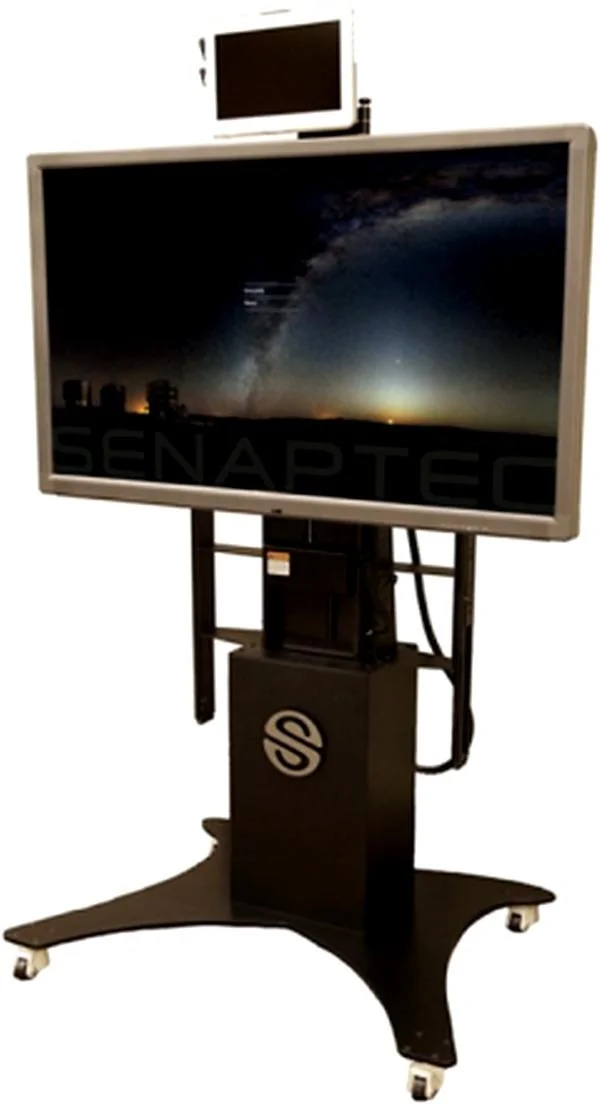
Senaptec Sensory Station (SS201) for visual ability testing

Visual ability was assessed using the Senaptec Sensory Station system (SS201, USA). This system has been widely used for evaluating the visual capabilities of athletes [23–25], with its reliability and validity established in previous studies [25, 26]. In line with the objectives of this study, three indicators were selected for testing: RT, EHC, and GNG. All tests were conducted by trained researchers following standardized procedures, and the results were automatically recorded by the system. The specific testing equipment is shown in Fig. 4, and the detailed procedures for each test are provided in Table 3.

**Table 3:** Detailed procedures for visual ability tests

Test Indicator	Starting Position and Initial Posture	Specific Requirements	Scoring Criteria	Test Content	Test Purpose
Reaction Time (RT)	The participant stands 60 cm from the tablet, with their eyes at the same horizontal level as the center of the screen.	The participant first selects their dominant hand. Two circles will appear on the screen, and the participant must touch the center of each circle with their index fingers. The circles will then turn green. Afterward, the circles will randomly turn red, and the participant must quickly raise the corresponding index finger and then lower it.	The test includes the reaction times for the dominant hand, non-dominant hand, and average reaction time, measured in milliseconds (ms). The faster the response, the better.	Measures the time taken by the participant to respond to a simple stimulus with a motor action.	The simple reaction time test reflects the athlete’s speed and efficiency in responding to basic visual stimuli.
Eye-Hand Coordination (EHC)	The athlete stands 60 cm from a large screen. The screen’s center line is aligned or slightly higher than the athlete’s arms, avoiding interference with peripheral vision.	A large display screen shows 8 columns and 10 rows of hollow circles. One of the circles will randomly turn blue-green, and the participant must click it. Afterward, another circle will appear at a random position. The goal is to click as many circles as possible within the specified time, as quickly and accurately as possible.	The test indicators include total time, average response time, central region response time, and peripheral region response time, all measured in milliseconds (ms). The smaller the value, the better.	Measures the participant’s hand speed in responding to rapidly changing targets through visual guidance.	Based on visual information acquisition, this test measures the athlete’s ability to process and integrate visual cues, guiding the body to respond quickly, coherently, and accurately. It reflects the coordination between the visual system and the motor execution system.
Decision-Making Ability (GNG)		The Senaptec large screen displays 8 columns of circles, similar to the eye-hand coordination task. Green or red dots will randomly appear on the circles. When a green dot appears, the athlete must quickly tap it. When a red dot appears, the athlete must refrain from tapping.	The test indicators include the overall score, with higher scores for correct taps, and lower scores for incorrect taps. The higher the correct score and the lower the incorrect score, the better.	Measures the participant’s ability to execute and inhibit visual-guided actions, and to respond with hand movements under “Go” and “No-Go” stimuli.	This test reflects the athlete’s ability to control response inhibition and cognitive flexibility, based on their ability to quickly and accurately locate targets.

#### Sport-specific performance assessment

Sport-specific performance was assessed using coordination quality [6], agility [7], and defensive reception success rate [12]. All tests were conducted on the same volleyball court under standardized conditions. Testing equipment, court layout, trial order, verbal instructions, and scoring procedures were kept consistent between the pre- and post-intervention assessments. Detailed test layouts, procedures, trial numbers, rest intervals, and scoring criteria are presented in Table 4. The coordination-quality outcome was automatically recorded by the FITLIGHT system. Defensive reception trials were recorded using a Casio EX-FH25 high-speed camera and independently evaluated in Kinovea by two experienced assessors blinded to group allocation and assessment time point.

**Table 4:** Operational procedures for sport-specific performance tests

Test Indicator	Test layout and procedure	Trials, rest interval, and scoring criteria	Test purpose
Agility Test	The agility test was conducted as a five-repetition shuttle-run field test. After a standardized 15-min warm-up, each participant started from the beginning mat and performed five shuttle runs toward designated test gates in response to a randomly presented light signal. Each gate was 1 m wide, and the distance from the starting mat to the gate was 4.5 m. The test required repeated acceleration, deceleration, stop-and-go movements, directional changes, and return movements.	Each participant completed two official trials, separated by a 3-min rest interval. The shortest completion time, recorded in seconds, was used for statistical analysis. The same court surface, route layout, start signal, verbal instructions, and timing procedure were used during both pre- and post-intervention testing.	Evaluates rapid acceleration, deceleration, directional change, and reactive movement ability under time pressure.
Coordination Quality	Coordination quality was assessed using a FITLIGHT-based light-response task. Eight LED light sensors were arranged in a semicircular configuration. For upper-limb responses, the lights were positioned approximately at forearm-length distance from the participant. For lower-limb responses, the participant stood in the center of the semicircular arrangement, with the distance from the feet to the lights corresponding approximately to the participant’s lower-limb length. Lights were activated randomly, and participants deactivated the illuminated lights as quickly as possible using the designated hand or foot.	Each test lasted 45 s, during which the average response time was automatically recorded by the FITLIGHT system. Each participant completed two official trials, separated by a 3-min rest interval. The best average response time was used for statistical analysis. Uniform instructions and demonstrations were provided before testing, and the same FITLIGHT configuration, testing procedure, and scoring criteria were used during pre- and post-intervention testing.	Evaluates visual stimulus response, upper- and lower-limb coordination, and continuous movement control.
Defensive Reception Success Rate	Defensive reception success rate was assessed using a volleyball-specific defensive reception test adapted from Hülsdünker et al. [12]. After a standardized warm-up and five familiarization trials, participants started from position 6 and received balls delivered by the same experienced coach toward randomized and balanced left, central, and right defensive zones. Ball speed was maintained at 65–75 km·h⁻¹ and monitored using a Bushnell Velocity Speed Gun.	Each participant completed 20 official trials in two blocks of 10, with 2 min of rest between blocks. A reception was scored as successful when the ball was controlled and directed into the designated setter area for a subsequent contact. Defensive reception success rate was calculated as successful receptions / 20 trials × 100%. Trials were video-recorded and evaluated using Kinovea.	Evaluates defensive reception performance in a standardized volleyball-specific situation.

### Statistical analysis

All analyses were performed using R software, version 4.4.1. Participants with complete baseline and post-intervention data were included, and no missing outcome data were imputed. Continuous variables are presented as mean ± standard deviation or median [Q1, Q3], according to data distribution. Normality and homogeneity of variance were assessed using the Shapiro–Wilk and Levene tests, respectively. Baseline comparisons were performed using independent-samples t-tests, Welch’s t-tests, or Mann–Whitney U tests, as appropriate.

Intervention effects were primarily examined using two-way repeated-measures ANOVA, with group as the between-subject factor, time as the within-subject factor, and the Group × Time interaction as the principal test of the intervention effect. Partial eta squared was reported as the effect size. Because only two time points were assessed, the sphericity assumption was not applicable.

Sensitivity analyses included between-group comparisons of improvement scores and baseline-adjusted ANCOVA. Improvement scores were calculated so that positive values indicated better performance. Between-group comparisons were conducted using independent-samples t-tests, Welch’s t-tests, or Mann–Whitney U tests, as appropriate. For parametric analyses, mean differences with 95% confidence intervals and Cohen’s d were reported. For Mann–Whitney U analyses, Hodges–Lehmann location shifts with 95% confidence intervals and Cliff’s delta were reported. ANCOVA models used the post-intervention value as the dependent variable, group as the independent variable, and the corresponding pre-intervention value as the covariate.

Defensive reception success rate was treated as the primary outcome, whereas the remaining outcomes were considered secondary or exploratory. Bonferroni correction was applied to secondary and exploratory outcomes. All tests were two-sided, and *p* < 0.05 was considered statistically significant.

## Results

### Baseline characteristics

Baseline characteristics and outcome measures are summarized in Table 5. A total of 26 participants were included in the complete-case analysis, with 13 participants in each group. No meaningful baseline imbalances were observed between groups. All analyzed participants completed the required intervention sessions, and no intervention-related adverse events were reported.

**Table 5:** Baseline characteristics and baseline outcome measures of participants

Section	Variable	Unit	EG (*n* = 13)	CG (*n* = 13)	*p* value
Participant characteristics	Age	years	20.50 ± 1.30	20.30 ± 1.30	0.698
	Height	cm	184.20 ± 6.10	183.60 ± 5.70	0.798
	Body mass	kg	76.40 ± 7.20	75.70 ± 6.80	0.801
	Training experience	years	6.80 ± 1.50	6.60 ± 1.40	0.728
Baseline outcome measures	Defensive reception success rate	%	55.30 ± 6.20	54.80 ± 5.90	0.835
	Reaction time	ms	305.80 ± 10.90	306.60 ± 11.40	0.856
	Eye–hand coordination	ms	533.64 [524.35, 569.29]	550.59 [531.18, 571.67]	0.626
	Decision-making ability	points	7.52 ± 0.88	7.46 ± 0.94	0.868
	Coordination quality	s	5.84 ± 0.25	5.82 ± 0.27	0.846
	Agility	s	18.01 [16.90, 19.12]	18.12 [17.34, 19.30]	0.457

Values are presented as mean ± standard deviation for approximately normally distributed variables and as median [Q1, Q3] for non-normally distributed variables. Eye–hand coordination and agility are presented as median [Q1, Q3]. Between-group comparisons were performed using independent-samples t-tests, Welch’s t-tests, or Mann–Whitney U tests, as appropriate. All values were derived from the same complete-case sample used in Table 6. *EG* Experimental group, *CG* Active-control group

### Intervention effects on visual abilities and sport-specific performance

As shown in Table 6, defensive reception success rate, the primary outcome, increased from 55.30 ± 6.20% to 61.05 ± 3.00% in the experimental group and from 54.80 ± 5.90% to 57.05 ± 4.50% in the active-control group. The experimental group showed a greater improvement than the active-control group, with a between-group difference in improvement of 3.50% points (95% CI, 1.50 to 5.50). A significant Group × Time interaction was observed, F(1, 24) = 13.053, *p* = 0.001, with a large effect size (partial η² = 0.352).

**Table 6:** Effects of the intervention on visual abilities and sport-specific performance

Outcome	Unit	EG Pre	EG Post	CG Pre	CG Post	Mean difference in improvement (95% CI)	Group × Time F (1, 24)	*p* value	Partial η²
Defensive reception success rate	%	55.30 ± 6.20	61.05 ± 3.00	54.80 ± 5.90	57.05 ± 4.50	3.50 (1.50, 5.50)	13.053	0.001	0.352
Reaction time	ms	305.80 ± 10.90	280.50 ± 7.80	306.60 ± 11.40	300.20 ± 10.60	18.90 (15.66, 22.14)	145.117	< 0.001	0.858
Eye–hand coordination	ms	548.70 ± 33.80	514.00 ± 18.00	551.20 ± 35.10	539.60 ± 33.00	23.10 (11.74, 34.46)	17.623	0.002	0.423
Decision-making ability	points	7.52 ± 0.88	7.76 ± 0.79	7.46 ± 0.94	7.53 ± 0.91	0.16 (0.05, 0.27)	9.273	0.030	0.279
Coordination quality	s	5.84 ± 0.25	5.31 ± 0.23	5.82 ± 0.27	5.71 ± 0.24	0.42 (0.34, 0.50)	114.660	< 0.001	0.827
Agility	s	17.95 ± 1.10	17.47 ± 0.68	18.18 ± 1.03	18.02 ± 0.91	0.32 (0.05, 0.59)	5.956	0.110	0.199

Pre- and post-intervention values are presented as mean ± standard deviation using the same complete-case sample as Table 5. For eye–hand coordination and agility, baseline values differ from Table 5because Table 5reports median [Q1, Q3]. The p value for the primary outcome was unadjusted, whereas secondary and exploratory p values were Bonferroni-adjusted. Improvement was coded so that positive values indicate better performance, and positive between-group differences favor the experimental group. *EG* Experimental group, *CG* Active-control group, *CI* Confidence interval

**Fig. 5:**
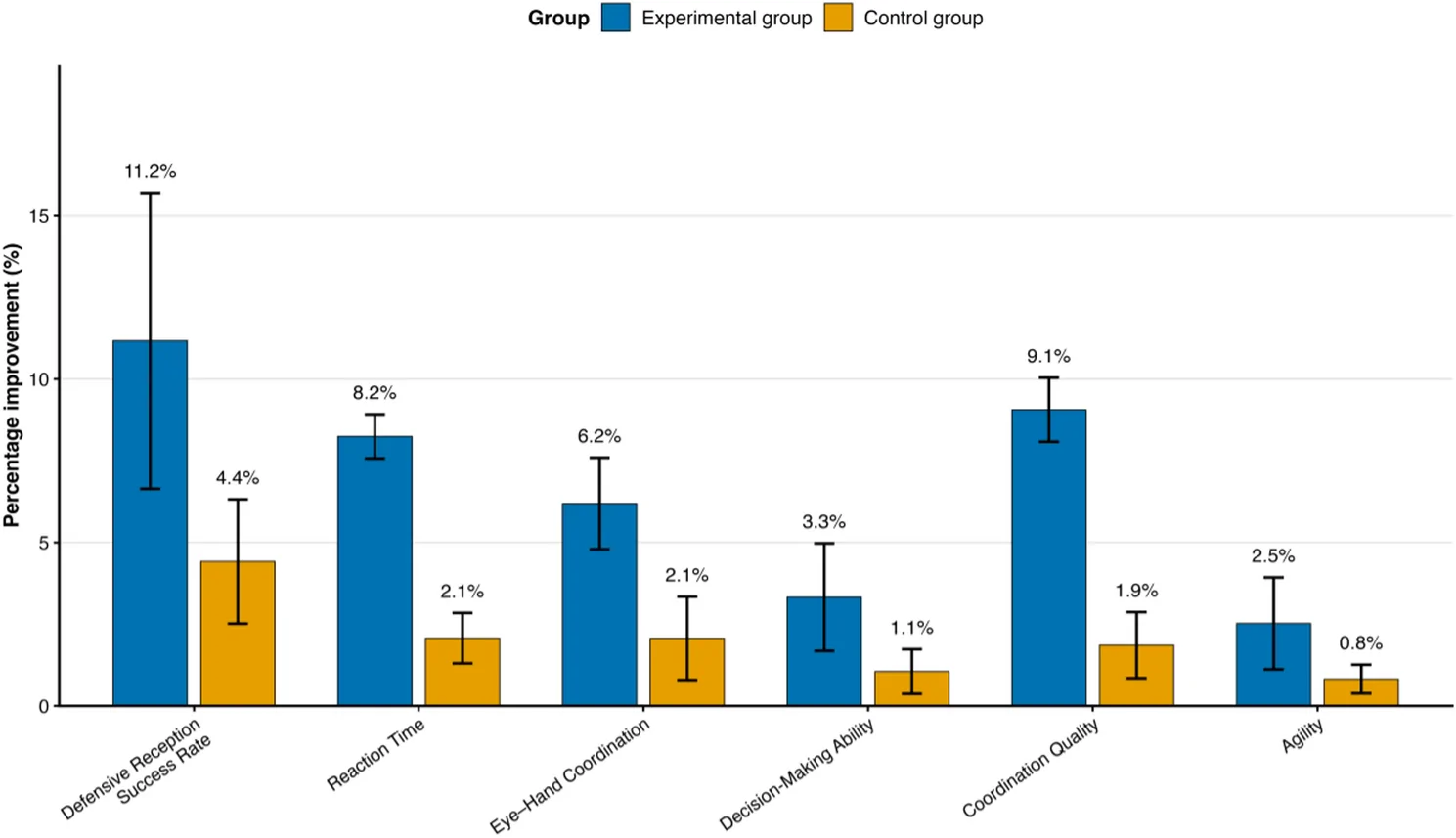
Percentage improvement in visual ability outcomes and sport-specific performance outcomes by group. Note. Bars represent mean percentage improvement, and error bars represent 95% confidence intervals. For reaction time, eye–hand coordination, coordination quality, and agility, improvement was calculated as pre-intervention value minus post-intervention value because lower values indicate better performance. For decision-making ability and defensive reception success rate, improvement was calculated as post-intervention value minus pre-intervention value because higher values indicate better performance. EG, experimental group; CG, active-control group

For the secondary and exploratory outcomes, significant Group × Time interactions after Bonferroni correction were observed for reaction time, eye–hand coordination, decision-making ability, and coordination quality, all favoring the experimental group. In contrast, agility did not remain significant after correction and was therefore interpreted as inconclusive. Detailed effect estimates are presented in Table 6, and full repeated-measures ANOVA results are provided in Supplementary Table S1 (Fig. 5). 

### Sensitivity analyses

Sensitivity analyses generally supported the primary findings. Improvement-score analyses indicated greater improvements in the experimental group for defensive reception success rate, reaction time, eye–hand coordination, decision-making ability, and coordination quality, whereas the evidence for agility remained inconclusive. The distributions of improvement scores are presented in Supplementary Figure S1.

Baseline-adjusted ANCOVA yielded effect estimates that were directionally consistent with those from the repeated-measures ANOVA and improvement-score analyses. Model diagnostics indicated that the ANCOVA assumptions were not fully satisfied for several outcomes, thereby reducing the robustness of the corresponding estimates. Accordingly, the ANCOVA findings were interpreted in conjunction with the primary and improvement-score analyses. Detailed improvement-score analyses, ANCOVA results, and model diagnostics are presented in Supplementary Tables S2–S4.

## Discussion

The main finding of this study was that, after 8 weeks of combined visual training, the experimental group showed greater improvements than the active-control group in selected visual ability and sport-specific performance outcomes. These findings suggest that combined stroboscopic and reaction-light training may provide additional benefits beyond matched volleyball-specific training performed under normal visual conditions. Importantly, these results should be interpreted not only according to statistical significance, but also in relation to effect-size magnitude, sensitivity analyses, model diagnostics, and statistical uncertainty.

From a statistical perspective, the evidence was strongest for outcomes that remained consistent across the repeated-measures ANOVA and the improvement-score sensitivity analyses. For the primary outcome, defensive reception success rate showed a significant Group × Time interaction with a large effect size (partial η² = 0.352), and a large between-group effect in the improvement-score sensitivity analysis (Cohen’s d = 1.417). This supports the cautious practical interpretation that the combined intervention may contribute to sport-specific performance transfer, rather than only improving isolated laboratory-based visual measures. Among the secondary or exploratory outcomes, reaction time and coordination quality showed particularly large effects, while eye-hand coordination and decision-making ability showed directionally consistent moderate-to-large improvements across the main and sensitivity analyses. These findings suggest potential benefits for rapid visual-motor processing and selected perceptual-motor abilities relevant to volleyball performance.

The improvements in reaction time, eye-hand coordination, and decision-making ability may be linked to increased visual information processing efficiency [21, 27]. One possible mechanism is that stroboscopic visual training may increase processing demands by intermittently limiting visual input, thereby encouraging athletes to extract key cues more efficiently and make adaptive adjustments [12]. In addition, reaction-light training reinforces rapid visual stimulus recognition, response selection, and action execution, which may enhance visual-motor coupling and coordination performance [16, 28]. Because the active-control group completed matched volleyball-specific tasks under normal visual conditions, the smaller magnitude of improvement in this group may reflect the absence of intermittent visual occlusion and randomized reaction-light stimuli, rather than differences in training duration or general task exposure.

These findings are broadly consistent with previous studies reporting that stroboscopic visual training can improve visuomotor reaction speed, perceptual processing, and sport-related visual-motor performance in athletes [7, 8, 12]. For example, prior volleyball-related studies have suggested that stroboscopic training may enhance reaction agility and visuomotor response speed, supporting the view that intermittent visual occlusion can increase perceptual demands and promote more efficient use of task-relevant visual information [7, 8]. Similarly, studies using reaction-light or FITLIGHT-based training have reported improvements in visual reaction time, movement initiation, and sport-specific skill performance [16, 17, 28]. The present findings extend this literature by combining stroboscopic visual restriction with randomized reaction-light stimuli in a volleyball-specific training context. This combined design may explain why the largest and most consistent effects were observed for outcomes closely related to rapid visual-motor processing and defensive performance.

The improvement in coordination quality suggests that combined visual training may positively influence the control of volleyball-specific movements. However, this finding should be interpreted with caution because the coordination-quality assessment involved light-based response tasks similar to those used during reaction-light training. Therefore, part of the observed improvement may reflect task-specific learning or test familiarization rather than a generalized improvement in coordination. This interpretation is consistent with previous work suggesting that perceptual-motor training is more effective when there is a strong match between training and assessment tasks [6]. In the present study, reaction-light training emphasized information recognition, movement adjustment, and body coordination under dynamic visual stimuli, while the coordination-quality assessment focused on body control and upper- and lower-limb coordination. The close alignment of these task demands may explain the large improvement observed in the experimental group.

The agility outcome requires more cautious interpretation. Although the repeated-measures ANOVA showed a significant Group × Time interaction for agility before correction, this effect did not remain significant after Bonferroni correction and was not confirmed by the Bonferroni-corrected improvement-score sensitivity analysis. In addition, the improvement-score effect size for agility was smaller (Cliff’s δ = 0.320) than those observed for reaction time, coordination quality, and defensive reception success rate, and the baseline-adjusted ANCOVA for agility was affected by unmet model assumptions. Taken together, these findings suggest that the agility result may be sensitive to analytical assumptions and should be regarded as preliminary rather than conclusive evidence of an intervention effect.

The improvement in defensive reception success rate provides preliminary and ecologically relevant evidence that the benefits of combined training may transfer from laboratory-based visual abilities to sport-specific performance. Volleyball defense requires athletes to rapidly identify incoming ball cues, predict ball trajectory, adjust positioning, and execute defensive actions. Therefore, this metric partially reflects the integration of visual processing and motor response [29, 30]. According to the modified perceptual training framework, transfer is more likely when training tasks resemble the competitive environment in perceptual information, action responses, and contextual features [31]. In the present study, stroboscopic training restricted visual input without altering the core volleyball task, which may have encouraged athletes to process key ball-flight information more efficiently. Meanwhile, reaction-light training involved randomized visual stimuli and defensive movement tasks, which may have supported rapid recognition, response initiation, and action execution.

Sensitivity analyses and model diagnostics provide important context for evaluating the strength of evidence. The improvement-score analyses supported the primary outcome and several secondary outcomes, increasing confidence that these findings were not dependent on a single repeated-measures ANOVA model. The baseline-adjusted ANCOVA results were also directionally consistent with the main analyses, but because some ANCOVA assumptions were not fully met, these results were interpreted as supportive rather than confirmatory. Overall, the evidence is strongest for outcomes that were consistent across the main analysis, multiple-testing correction, and improvement-score sensitivity analysis, whereas outcomes with inconsistent sensitivity results or unmet model assumptions should be interpreted more cautiously.

Statistical uncertainty should also be considered. The final complete-case sample included 13 participants per group, and the sample-size sensitivity analysis indicated that the study was powered to detect only large between-group effects. Therefore, large and consistent effects should be considered more informative in the present dataset than small or unstable effects. Conversely, non-significant or inconsistent findings should not be interpreted as definitive evidence of no effect. Confidence intervals provide additional information about the precision of the observed effects; wider intervals indicate greater uncertainty and the need for replication in larger samples. Accordingly, the present findings should be viewed as promising but preliminary evidence that combined stroboscopic and reaction-light training may improve selected visual and sport-specific performance outcomes in collegiate volleyball players.

### Practical implications

The results of this study have practical implications for volleyball training. Volleyball competitions are characterized by significant time pressure and situational uncertainty, especially during defense and serve-receive situations. Athletes need to quickly identify incoming ball cues, make judgments, adjust their movements, and execute technical actions. Therefore, training methods that target both visual information processing and quick reaction abilities are of practical value in improving sport-specific performance. The combined visual training approach used in this study integrates stroboscopic visual training and reaction light training. The former may help athletes practice extracting task-relevant information under restricted visual input, while the latter may support rapid recognition of external stimuli, response initiation, and action execution. These two training methods may complement each other in terms of training demands. The results of this study suggest that this training model may improve selected visual ability outcomes and sport-specific performance outcomes. Given its short implementation time, relatively simple operation, and ease of integration into regular volleyball training, this approach has promising practical application potential. Based on this, coaches can tailor the use of visual training equipment to match specific tasks and needs in training, thereby enhancing the relevance, efficiency, and scientific basis of the training process.

Because neurophysiological measures were not collected in the present study, the proposed mechanisms should be interpreted as possible explanations rather than confirmed mechanisms.

### Study limitations and future directions

Several limitations should be acknowledged. First, the sample size was relatively small, and the final analysis was based on complete cases rather than an intention-to-treat approach, which may limit statistical power and introduce potential attrition bias. Second, the trial was retrospectively registered; therefore, the primary outcome and statistical analysis framework were clarified during manuscript revision rather than fully prespecified before participant recruitment. Third, because of the field-based nature of the intervention, participant and trainer blinding was not feasible, and complete assessor blinding could not be fully guaranteed. Fourth, some outcomes, particularly coordination quality, may have been influenced by task-specific learning because the assessment involved light-based response tasks similar to the training tasks. Fifth, retention testing was not conducted, so the durability of the training effects remains unclear. Finally, the participants were collegiate male volleyball players, which may limit the generalizability of the findings to other populations. Future studies should include larger and more diverse samples, adopt prospective registration, and examine long-term training effects.

## Conclusions

In conclusion, compared with matched volleyball-specific active-control training under normal visual conditions, eight weeks of combined stroboscopic glasses and reaction-light training may provide additional benefits for improving defensive reception success rate and selected visual ability outcomes in collegiate male volleyball players. However, the findings should be interpreted cautiously because of the small complete-case sample, retrospective trial registration, possible task-specific learning, lack of quantified inter-rater reliability for defensive reception scoring, and absence of retention testing. The agility finding was not confirmed by the corrected sensitivity analysis and should be regarded as preliminary.

## Supplementary Information

Supplementary Material 1.

Supplementary Material 2.

## Data Availability

The datasets generated and/or analysed during the current study are available from the corresponding author on reasonable request.

## Abbreviations

EG: Experimental Group
CG: Control Group
SVT: Stroboscopic Visual Training
RT: Reaction Time
EHC: Eye–Hand Coordination
GNG: Go/No-Go
CI: Confidence Interval
