# Supplementary material for: Effects of combined stroboscopic glasses and reaction light training on visual abilities and sport-specific performance in collegiate male volleyball players
Source: BMC Sports Sci Med Rehabil. 2026 Aug 7;18:555. doi: 10.1186/s13102-026-01957-6 (PMC13737681; doi:10.1186/s13102-026-01957-6)
Supplement: Supplementary file 1 [file 13102_2026_1957_MOESM1_ESM.docx]

CONSORT 2010 checklist of information to include when reporting a randomised trial*

Manuscript: Effects of Combined Stroboscopic Glasses and Reaction Light Training on Visual Abilities and Sport-Specific Performance in Collegiate Male Volleyball Players

| **Section/Topic** | **Item No** | **Checklist item** | **Reported on page No** |
| --- | --- | --- | --- |
| Title and abstract | | | |
|  | 1a | Identification as a randomised trial in the title | Not in title; randomized controlled pre-post design reported in Methods (p. 5). |
|  | 1b | Structured summary of trial design, methods, results, and conclusions (for specific guidance see CONSORT for abstracts) | Abstract, pp. 1-2. |
| Introduction | | | |
| Background and objectives | 2a | Scientific background and explanation of rationale | Introduction, pp. 2-5. |
|  | 2b | Specific objectives or hypotheses | Objectives and hypothesis, p. 5. |
| Methods | | | |
| Trial design | 3a | Description of trial design (such as parallel, factorial) including allocation ratio | Study Design, p. 5; Figure 1, p. 6. |
|  | 3b | Important changes to methods after trial commencement (such as eligibility criteria), with reasons | Not reported; no important changes after trial commencement stated. |
| Participants | 4a | Eligibility criteria for participants | Participants, inclusion/exclusion criteria, pp. 6-7. |
|  | 4b | Settings and locations where the data were collected | Participants from Hunan Normal University; testing/training settings described in Methods, pp. 6-19. |
| Interventions | 5 | The interventions for each group with sufficient details to allow replication, including how and when they were actually administered | Intervention Protocol, Table 1, and Figures 2-3, pp. 8-12. |
| Outcomes | 6a | Completely defined pre-specified primary and secondary outcome measures, including how and when they were assessed | Visual ability and sport-specific outcomes, Tables 2-3, pp. 12-19; assessment timing, pp. 5-7. |
|  | 6b | Any changes to trial outcomes after the trial commenced, with reasons | Not reported; no outcome changes after trial commencement stated. |
| Sample size | 7a | How sample size was determined | Post hoc/sensitivity power analysis reported, pp. 21 and 32-34; a priori sample-size determination not explicitly reported. |
|  | 7b | When applicable, explanation of any interim analyses and stopping guidelines | Not applicable; no interim analyses or stopping guidelines reported. |
| Randomisation: |  |  |  |
| Sequence generation | 8a | Method used to generate the random allocation sequence | Random assignment reported, pp. 5-7; random sequence generation method not explicitly reported. |
|  | 8b | Type of randomisation; details of any restriction (such as blocking and block size) | Two-group allocation reported in Figure 1, p. 6; restriction/blocking not reported. |
| Allocation concealment mechanism | 9 | Mechanism used to implement the random allocation sequence (such as sequentially numbered containers), describing any steps taken to conceal the sequence until interventions were assigned | Not reported. |
| Implementation | 10 | Who generated the random allocation sequence, who enrolled participants, and who assigned participants to interventions | Not reported. |
| Blinding | 11a | If done, who was blinded after assignment to interventions (for example, participants, care providers, those assessing outcomes) and how | Not reported. |
|  | 11b | If relevant, description of the similarity of interventions | Control group completed same/highly similar tasks without stroboscopic glasses/reaction lights, pp. 8 and 10-11. |
| Statistical methods | 12a | Statistical methods used to compare groups for primary and secondary outcomes | Statistical Analysis, pp. 20-21. |
|  | 12b | Methods for additional analyses, such as subgroup analyses and adjusted analyses | Sensitivity analyses and robustness analyses, pp. 20-21 and 32-34; subgroup analyses not reported. |
| Results | | | |
| Participant flow (a diagram is strongly recommended) | 13a | For each group, the numbers of participants who were randomly assigned, received intended treatment, and were analysed for the primary outcome | Participant flow described in Figure 1 and Participants section, pp. 6-7. |
|  | 13b | For each group, losses and exclusions after randomisation, together with reasons | Four withdrawals described in Participants/Figure 1, pp. 6-7. |
| Recruitment | 14a | Dates defining the periods of recruitment and follow-up | Not reported. |
|  | 14b | Why the trial ended or was stopped | Trial completed the planned 8-week intervention; early stopping not reported, pp. 5-8. |
| Baseline data | 15 | A table showing baseline demographic and clinical characteristics for each group | Baseline characteristics, Table 4, pp. 21-23. |
| Numbers analysed | 16 | For each group, number of participants (denominator) included in each analysis and whether the analysis was by original assigned groups | Final analysed sample reported as EG n=13 and CG n=13, pp. 6-7 and 21; analyses by assigned group implied. |
| Outcomes and estimation | 17a | For each primary and secondary outcome, results for each group, and the estimated effect size and its precision (such as 95% confidence interval) | Main outcomes and effect sizes, Table 5 and Figures 5-7, pp. 23-28; within-group and robustness results, Tables 6-7, pp. 29-34. |
|  | 17b | For binary outcomes, presentation of both absolute and relative effect sizes is recommended | Not applicable; no binary outcomes reported. |
| Ancillary analyses | 18 | Results of any other analyses performed, including subgroup analyses and adjusted analyses, distinguishing pre-specified from exploratory | Supplementary within-group analyses and G*Power-equivalent robustness analysis, pp. 28-34. |
| Harms | 19 | All important harms or unintended effects in each group (for specific guidance see CONSORT for harms) | Not formally reported; four withdrawals due to personal reasons or minor/unrelated injuries noted, p. 6. |
| Discussion | | | |
| Limitations | 20 | Trial limitations, addressing sources of potential bias, imprecision, and, if relevant, multiplicity of analyses | Study limitations and future directions, pp. 39-40. |
| Generalisability | 21 | Generalisability (external validity, applicability) of the trial findings | Generalisability discussed in limitations, pp. 39-40. |
| Interpretation | 22 | Interpretation consistent with results, balancing benefits and harms, and considering other relevant evidence | Discussion and interpretation, pp. 35-39. |
| Other information | | |  |
| Registration | 23 | Registration number and name of trial registry | Page 2. Trial registration: ISRCTN Registry ISRCTN10147263, registered on 27 May 2026. Retrospectively registered. |
| Protocol | 24 | Where the full trial protocol can be accessed, if available | Not reported; full trial protocol availability not stated. |
| Funding | 25 | Sources of funding and other support (such as supply of drugs), role of funders | Funding statement, p. 40: no external funding. |

Citation: Schulz KF, Altman DG, Moher D, for the CONSORT Group. CONSORT 2010 Statement: updated guidelines for reporting parallel group randomised trials. BMC Medicine. 2010;8:18.
© 2010 Schulz et al. This is an Open Access article distributed under the terms of the Creative Commons Attribution License (<http://creativecommons.org/licenses/by/2.0>), which permits unrestricted use, distribution, and reproduction in any medium, provided the original work is properly cited.

*We strongly recommend reading this statement in conjunction with the CONSORT 2010 Explanation and Elaboration for important clarifications on all the items. If relevant, we also recommend reading CONSORT extensions for cluster randomised trials, non-inferiority and equivalence trials, non-pharmacological treatments, herbal interventions, and pragmatic trials. Additional extensions are forthcoming: for those and for up-to-date references relevant to this checklist, see [www.consort-statement.org](http://www.consort-statement.org/).
