# Supplementary material for: Effects of combined stroboscopic glasses and reaction light training on visual abilities and sport-specific performance in collegiate male volleyball players
Source: BMC Sports Sci Med Rehabil. 2026 Aug 7;18:555. doi: 10.1186/s13102-026-01957-6 (PMC13737681; doi:10.1186/s13102-026-01957-6)
Supplement: Supplementary file 2 [file 13102_2026_1957_MOESM2_ESM.docx]

# Supplementary Material

**Supplementary Table S1. Full repeated-measures ANOVA results**

| Outcome | Effect | df | F | p value | Adjusted p value | Partial η² |
| --- | --- | --- | --- | --- | --- | --- |
| Defensive reception success rate | Group main effect | 1, 24 | 1.365 | 0.254 | — | 0.054 |
| Defensive reception success rate | Time main effect | 1, 24 | 68.197 | <0.001 | — | 0.740 |
| Defensive reception success rate | Group × Time interaction | 1, 24 | 13.053 | 0.001 | — | 0.352 |
| Reaction time | Group main effect | 1, 24 | 6.729 | 0.016 | — | 0.219 |
| Reaction time | Time main effect | 1, 24 | 408.237 | <0.001 | — | 0.944 |
| Reaction time | Group × Time interaction | 1, 24 | 145.117 | <0.001 | <0.001 | 0.858 |
| Eye–hand coordination | Group main effect | 1, 24 | 1.429 | 0.244 | — | 0.056 |
| Eye–hand coordination | Time main effect | 1, 24 | 70.796 | <0.001 | — | 0.747 |
| Eye–hand coordination | Group × Time interaction | 1, 24 | 17.623 | <0.001 | 0.002 | 0.423 |
| Decision-making ability | Group main effect | 1, 24 | 0.168 | 0.686 | — | 0.007 |
| Decision-making ability | Time main effect | 1, 24 | 33.491 | <0.001 | — | 0.583 |
| Decision-making ability | Group × Time interaction | 1, 24 | 9.273 | 0.006 | 0.030 | 0.279 |
| Coordination quality | Group main effect | 1, 24 | 3.979 | 0.058 | — | 0.142 |
| Coordination quality | Time main effect | 1, 24 | 266.240 | <0.001 | — | 0.917 |
| Coordination quality | Group × Time interaction | 1, 24 | 114.660 | <0.001 | <0.001 | 0.827 |
| Agility | Group main effect | 1, 24 | 1.146 | 0.295 | — | 0.046 |
| Agility | Time main effect | 1, 24 | 23.137 | <0.001 | — | 0.491 |
| Agility | Group × Time interaction | 1, 24 | 5.956 | 0.022 | 0.110 | 0.199 |

## *Note. df, degrees of freedom; partial η², partial eta squared. The Group × Time interaction was used as the principal test of the intervention effect. For the primary outcome, defensive reception success rate, the unadjusted p value was interpreted as the main confirmatory test. For secondary or exploratory outcomes, Bonferroni-adjusted p values were calculated for the Group × Time interaction across the five secondary or exploratory outcomes. Adjusted p values were not applied to group main effects or time main effects.*

**Supplementary Figure S1. Between-group comparison of improvement** **scores.**

*Note. Boxplots show the distribution of improvement scores in the experimental and active-control groups. The central line represents the median, the box represents the interquartile range, whiskers represent values within 1.5 times the interquartile range, and diamonds represent the mean. Positive values indicate greater improvement.*

**Supplementary Table S2. Improvement-score sensitivity analyses**

| **Outcome** | **Unit** | **EG improvement** | **CG improvement** | **Statistical method** | **Between-group estimate (95% CI)** | **Statistic** | **p value** | **Effect size** |
| --- | --- | --- | --- | --- | --- | --- | --- | --- |
| Defensive reception success rate | % | 5.75 ± 3.20 | 2.25 ± 1.40 | Welch independent-samples t-test | Mean difference: 3.50 (1.45, 5.55) | t = 3.613 | 0.002 | Cohen’s d = 1.417 |
| Reaction time | ms | 26.13 [24.40, 27.22] | 4.44 [3.29, 9.19] | Mann–Whitney U test | Hodges–Lehmann shift: 20.17 (15.69, 23.01) | U = 169.000 | <0.001 | Cliff’s δ = 1.000 |
| Eye–hand coordination | ms | 27.66 [23.32, 44.32] | 5.36 [4.85, 15.48] | Mann–Whitney U test | Hodges–Lehmann shift: 20.94 (16.75, 33.10) | U = 148.000 | 0.006 | Cliff’s δ = 0.751 |
| Decision-making ability | points | 0.20 [0.11, 0.39] | 0.05 [0.02, 0.11] | Mann–Whitney U test | Hodges–Lehmann shift: 0.13 (0.03, 0.28) | U = 138.000 | 0.030 | Cliff’s δ = 0.633 |
| Coordination quality | s | 0.54 [0.48, 0.61] | 0.05 [0.03, 0.17] | Mann–Whitney U test | Hodges–Lehmann shift: 0.44 (0.33, 0.51) | U = 169.000 | <0.001 | Cliff’s δ = 1.000 |
| Agility | s | 0.35 [0.05, 0.90] | 0.08 [0.06, 0.28] | Mann–Whitney U test | Hodges–Lehmann shift: 0.25 (−0.04, 0.62) | U = 111.500 | 0.830 | Cliff’s δ = 0.320 |

*Note. Improvement scores were calculated so that positive values indicated better performance. Values are presented as mean ± standard deviation for Welch’s t-test and as median [Q1, Q3] for Mann–Whitney U tests. For Mann–Whitney U analyses, the between-group estimate represents the Hodges–Lehmann location shift between the experimental group and the active-control group, with its 95% confidence interval. For Welch’s t-test, the between-group estimate represents the mean difference. The p value for the primary outcome is unadjusted; p values for secondary and exploratory outcomes are Bonferroni-adjusted. EG, experimental group; CG, active-control group; CI, confidence interval.*

**Supplementary Table S3. Baseline-adjusted ANCOVA results**

| **Outcome** | **Unit** | **Adjusted difference EG − CG (95% CI)** | **SE** | **df** | **t** | **p value** | **R²** | **Adjusted R²** |
| --- | --- | --- | --- | --- | --- | --- | --- | --- |
| Defensive reception success rate | % | 3.69 (2.99, 4.39) | 0.338 | 23.00 | 10.924 | <0.001 | 0.963 | 0.959 |
| Reaction time | ms | −19.07 (−21.72, −16.43) | 1.280 | 23.00 | −14.905 | <0.001 | 0.947 | 0.942 |
| Eye–hand coordination | ms | −23.81 (−32.11, −15.51) | 4.013 | 23.00 | −5.935 | <0.001 | 0.887 | 0.877 |
| Decision-making ability | points | 0.17 (0.07, 0.26) | 0.047 | 23.00 | 3.514 | 0.009 | 0.981 | 0.979 |
| Coordination quality | s | −0.42 (−0.49, −0.34) | 0.036 | 23.00 | −11.518 | <0.001 | 0.917 | 0.910 |
| Agility | s | −0.38 (−0.53, −0.23) | 0.073 | 23.00 | −5.251 | <0.001 | 0.956 | 0.952 |

*Note. ANCOVA models specified the post-intervention value as the dependent variable, group as the independent variable, and the corresponding pre-intervention value as the covariate. Negative adjusted differences favor the experimental group for outcomes in which lower values indicate better performance, including reaction time, eye–hand coordination, coordination quality, and agility. Positive adjusted differences favor the experimental group for outcomes in which higher values indicate better performance, including decision-making ability and defensive reception success rate. The p value for the primary outcome is unadjusted; p values for secondary and exploratory outcomes are Bonferroni-adjusted.EG, experimental group; CG, active-control group; CI, confidence interval.*

**Supplementary Table S4. ANCOVA assumption checks**

| **Outcome** | **Unit** | **Group × baseline p** | **Homogeneity of regression slopes** | **Residual Shapiro–Wilk p** | **Residual normality** | **Residual Levene p** | **Homoscedasticity** |
| --- | --- | --- | --- | --- | --- | --- | --- |
| Defensive reception success rate | % | <0.001 | Not satisfied | 0.145 | Satisfied | 0.675 | Satisfied |
| Reaction time | ms | 0.130 | Satisfied | 0.893 | Satisfied | 0.081 | Satisfied |
| Eye–hand coordination | ms | 0.002 | Not satisfied | 0.097 | Satisfied | 0.092 | Satisfied |
| Decision-making ability | points | 0.151 | Satisfied | 0.112 | Satisfied | 0.080 | Satisfied |
| Coordination quality | s | 0.911 | Satisfied | 0.468 | Satisfied | 0.782 | Satisfied |
| Agility | s | <0.001 | Not satisfied | 0.042 | Not satisfied | 0.938 | Satisfied |

***Note.*** *The homogeneity of regression slopes assumption was assessed using the Group × baseline interaction. Residual normality was assessed using the Shapiro–Wilk test, and homoscedasticity was assessed using Levene’s test.*
